# TSPO Expression and [18F]DPA‐714 PET/CT Imaging as Pathogenetic and Diagnostic Biomarkers in Symptomatic Stages of Skeletal Muscle Fiber Degeneration in SOD1‐G93A ALS Mice

**DOI:** 10.1096/fj.202502450R

**Published:** 2025-10-14

**Authors:** Serenella Anzilotti, Nunzia De Iesu, Sara Gargiulo, Noemi Di Muraglia, Annunziata Gaetana Cicatiello, Monica Dentice, Mariarosaria Panico, Sandra Albanese, Lucio Annunziato, Marco Salvatore, Giuseppe Pignataro, Sabina Pappatà

**Affiliations:** ^1^ Department of Human Sciences and Quality of Life Promotion San Raffaele University Rome Italy; ^2^ IRCCS SYNLAB SDN Naples Italy; ^3^ Siena Italy; ^4^ Division of Pharmacology, Department of Neuroscience, School of Medicine University of Naples “Federico II” Naples Italy; ^5^ Department of Clinical Medicine and Surgery University of Naples “Federico II” Naples Italy; ^6^ Institute of Biostructures and Bioimaging National Research Council Naples Italy

**Keywords:** 18F‐DPA‐714, amyotrophic lateral sclerosis, macrophages, mitochondrial fission, positron emission tomography, skeletal muscle, TSPO

## Abstract

Emerging evidence highlights the involvement of skeletal muscle in the pathogenesis of amyotrophic lateral sclerosis (ALS), through mechanisms involving inflammation and mitochondrial dysfunction in skeletal muscle fibers. The 18 kDa translocator protein (TSPO) is primarily expressed on the outer mitochondrial membrane, is implicated in inflammation, and serves as both a biomarker and a therapeutic target for neuroinflammation. This study investigated whether PET imaging targeting the TSPO, immunohistochemistry, and confocal microscopy can characterize skeletal muscle inflammation and muscular fiber damage in SOD1‐G93A ALS transgenic mice. High‐resolution PET/CT imaging with [18F]DPA‐714 was employed to assess TSPO expression in the triceps brachii of SOD1‐G93A mice at mild (age range: 98–112 days; Clinical Score (CS) range:1–1.5) and moderate–severe (age range: 120–137 days; CS range: 2–4) symptomatic stages. To support PET data, TSPO was analyzed by immunohistochemistry and confocal microscopy in the triceps skeletal muscle obtained from mild and moderate–severe SOD1‐G93A mice. Inflammatory and anti‐inflammatory macrophage cells in skeletal muscle tissues were detected by immunofluorescence. PET/CT revealed a progressive, significant increase of [18F]DPA‐714 uptake in SOD1‐G93A triceps brachii in mild and moderate–severe stages. Immunohistochemistry and confocal microscopy confirmed increased TSPO expression in the degenerating muscle fibers and in infiltrating macrophage cells. In vivo studies of TSPO expression in ALS‐affected skeletal muscles may provide valuable insights into muscle inflammation and mitochondrial involvement during disease progression. In addition, TSPO and PET/CT imaging with [18F]DPA‐714 might represent a noninvasive and promising diagnostic biomarker for detecting early muscle pathology in ALS.

## Graphical abstract

In the first step, PET imaging with the TSPO radioligand was performed in SOD1‐G93A mice at two disease stages: mild and moderate/severe. Subsequent analyses were conducted using immunohistochemistry and confocal microscopy to confirm TSPO expression in the triceps muscles of SOD1‐G93A mice. Both PET imaging and immunohistochemical analyses confirmed an increased TSPO expression already at the mild stages of the disease. Confocal microscopy further demonstrated that TSPO immunoreactivity colocalized with infiltrating macrophage cells and with mitochondrial fission marker.

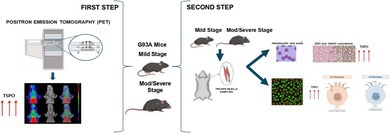

**Abbreviations**
ALS: amyotrophic lateral sclerosis
CNS: central nervous system
CS: clinical score
CSA: cross section area
CT: computed tomography
DPA: N,N‐diethyl‐2‐(2‐(4‐(2‐fluoroethoxy)phenyl)‐5,7‐dimethylpyrazolo[1,5‐a]pyrimidin‐3‐yl)acetamide
DRP1: dynamin‐related protein 1
FDG: fluorodeoxyglucose
HE: Hematoxylin/Eosin
MFN: mitofusion
MTT: thiazolyl blue tetrazolium bromide
OMM: outer mitochondrial membrane
PET: positron emission tomography
SDH: succinic dehydrogenase
SUV: standardized uptake value
TSPO: 18‐kDa translocator protein
VOI: spherical volume of interest
WT: wild type

## 1 Introduction

Amyotrophic lateral sclerosis (ALS) is a progressive neurodegenerative disorder characterized by the selective degeneration of motor neurons, ultimately resulting in muscle weakness, atrophy, and respiratory failure [1]. While the pathogenesis of ALS remains complex and multifactorial, mounting evidence highlights the pivotal role of inflammation and mitochondrial dysfunction in both the central and peripheral nervous systems [2, 3, 4]. Historically, research has primarily focused on neuroinflammation within the brain and spinal cord [5, 6]. However, more recent studies suggest that peripheral tissues, particularly skeletal muscles, also undergo significant inflammatory alterations during disease progression [7]. Peripheral inflammation in ALS is principally characterized by the activation of local immune cells, elevated levels of pro‐inflammatory cytokines, and the infiltration of macrophages into denervated skeletal muscles [8, 9]. This inflammatory cascade exacerbates muscle degeneration, thereby accelerating disease progression and aggravating functional decline [10]. Moreover, recent findings have identified mitochondrial defects in muscle as one of the earliest pathological events associated with ALS [11]. Notably, recent investigations have, for the first time, demonstrated that cholesterol metabolism and transport are compromised in ALS‐affected skeletal fibers before the onset of clinical symptoms [12]. This metabolic dysregulation leads to cholesterol accumulation within muscle tissue, which positively correlates with the severity of motor dysfunction observed in ALS patients [12]. Among the molecular markers associated with these alterations, the 18‐kDa translocator protein (TSPO) has emerged as a potential key regulator. TSPO is a mitochondrial protein primarily involved in cholesterol transport and steroid biosynthesis; however, its functional scope extends beyond metabolic pathways to include the modulation of inflammatory responses [13]. Furthermore, emerging evidence suggests that TSPO is not simply a passive indicator of inflammation but may also play an active role in regulating cellular inflammatory and metabolic processes that mediate mitochondrial quality control and oxidative stress [14]. These findings imply that TSPO may act as a critical mediator in the crosstalk between mitochondrial function and inflammation, thereby influencing disease pathogenesis. Positron emission tomography (PET) using selective radioligands for TSPO has been successfully applied for in vivo imaging of CNS neuroinflammation in clinical neurological diseases [15] and animal models of brain diseases [16]. In addition, TSPO PET radioligands have been used to detect peripheral inflammation, as shown by TSPO upregulation occurring in activated macrophages and other peripheral immune cells [17]. We have previously demonstrated increased microglial activation, associated with TSPO expression, in the brainstem of symptomatic ALS mice using [18F]DPA‐714 PET/CT [18]. In vivo studies of TSPO expression in ALS‐affected skeletal muscles may provide valuable insights into muscle inflammation and mitochondrial involvement during disease progression. However, TSPO expression in ALS‐affected muscles remains poorly explored. The present study aimed to investigate and characterize the TSPO expression and its cellular localization in skeletal muscles of SOD1‐G93A mice at different clinical stages using micro‐PET/CT imaging with [18F]DPA‐714 and immunohistochemistry. Specifically, we used light microscopy and confocal double immunofluorescence studies to characterize the colocalization of TSPO expression with macrophages and mitochondrial markers. The findings derived from this study provide critical insights into the complex relationship between peripheral inflammation, mitochondrial muscle degeneration, and ALS progression, thereby offering alternative options for the development of innovative diagnostic and therapeutic strategies to counteract this debilitating disorder.

## 2 Methods

### 2.1 Animal Model and Study Design

B6SJL‐TgN SOD1/G93A(+)1Gur mice expressing a high copy number of mutant human SOD1 with a Gly93Ala substitution (G93A) and B6SJL‐TgN (SOD1) 2Gur mice (RRID:IMSR_JAX:002726) expressing wild‐type human SOD1 (WT) were obtained from Jackson Laboratories (RRID:IMSR_JAX:002300). This model was chosen for experiments as it remains the only validated mouse model of ALS for preclinical study according to the ALS Therapy Development Institute and reproduces much of the pathogenesis and pathology of clinical disease. In vivo imaging PET‐CT was performed on hemizygous transgenic male B6 SJL‐Tg[SOD1*G93A]1Gur/J mice (SOD1‐G93A; stock number 002726) (RRID:IMSR_JAX:002300), and their control counterpart hemizygous transgenic B6SJL‐Tg(SOD1)2Gur/J mice (RRID:IMSR_JAX:002726) (WT SOD1; stock number 002297) [18]. We conducted secondary exploratory research using part of the experimental data collected for a previous study [18] to address a novel scientific question on the feasibility of TSPO imaging in skeletal muscles of the mouse model of ALS using an integrative analytical perspective. The aim of this preliminary investigation was to gain further insights for a deeper understanding of the phenotyping data obtained from this animal model, thus reducing the number of animals to obtain valid scientific results according to the 3Rs. Additionally, immunohistochemistry studies were performed on a cohort of mice bred from the same progenitors. For this, transgenic animals have been crossed with background‐matched B6SJL wild‐type females, and selective breeding has maintained each transgene in the homozygous state. All transgenic mice were identified by analyzing DNA extracts from tail tips by PCR as previously described [19]. Mice were group‐housed in the same experimental room and maintained under standard controlled light/dark cycles, environmental temperature and humidity conditions, and had libitum access to standard diet and filtered water. Supportive therapies were implemented into the animal care and use program by a qualified and experienced veterinarian to minimize the suffering and discomfort of the mice according to the 3Rs [18].

Disease onset and progression in SOD1‐G93A mice were clinically assessed three times a week starting from 50 days of age as described in detail elsewhere [18]. For human endpoints, a clinical score (CS) of 4 was set.

### 2.2 Ethics Statement

All in vivo experiments were carried out according to the provisions of the European Communities Council Directive (86/609/CEE; 2010/63/EU) and of the Italian Ministry of Health (D.L. 116/92 and subsequent amending DL 26/2016). The experiments were formally approved by the Animal Care Committee of the University “Federico II” of Naples, Italy, and authorized by the Italian Ministry of Health. The guidelines of the National Institutes of Health (NIH) were followed for the design and execution of in vivo experiments.

### 2.3 In Vivo PET‐CT Imaging of [18F]DPA714

Nine symptomatic SOD1‐G93A mice (age range: 98–137 days, mean ± SD 117 ± 14 days; CS range: 1–4) and four congenic controls WT SOD1 mice (age range: 89–137; mean ± SD 117 ± 22.6 days; CS: 0) were examined by in vivo imaging. The nine symptomatic SOD1‐G93A mice were divided into two groups according to the clinical severity: mild (*n* = 4; CS range: 1–1.5; age range: 98–112 days, mean ± SD 116 ± 13 days) and moderate–severe (*n* = 5; CS range: 2–4; age range: 120–137 days, mean ± SD 127 ± 8). Groups were assigned based on genotype without randomization, and no blinding was applied. PET/CT imaging was performed using a high‐resolution PET scanner (GE Healthcare eXplore Vista; resolution 1.8 mm FWHM/200 μm, sensitivity 4.2% ACS) in mice anesthetized with 2% isoflurane plus 2 L/min oxygen. PET images were acquired in dynamic mode (frame sequence: 6 × 5 min) starting 20 min after tail vein injection of [18F]DPA‐714 (5.55–7.00 MBq, specific radioactivity 200–800 GBq/μmol) [20] for a duration of 30 min. Images were processed using a 2D FORE/3D OSEM iterative algorithm (voxel size 0.3875 × 0.3875 × 0.775 mm), including random, scatter, dead time, and decay correction. Data analysis was performed on PET frames summed from 20 to 50 min after intravenous injection of the radiotracer. Spherical volumes of interest (VOIs) (radius: 1 mm) were placed on the right and left triceps brachii and on the associative frontal cortex across consecutive slices using PMOD software (PMOD Software RRID:SCR_016547). Anatomical information from CT was used to optimize the VOIs placement, thus improving the accuracy of radiotracer uptake assessment in skeletal muscle and brain. [18F]DPA‐714 standardized uptake value (SUV) values were calculated for the associative frontal cortex and right and left triceps VOIs. The SUV values from the right and left triceps were averaged. SUV ratios (SUVR) were then generated by normalizing the averaged triceps SUV values to those of the frontal association cortex. This normalization was performed to reduce interanimal variability in radiotracer uptake. The frontal association cortex was used for normalization because this region is unaffected in SOD1‐G93A, as previously shown [18].

### 2.4 Tissue Processing, Immunostaining, and Confocal Immunofluorescence

For immunohistochemistry and confocal microscopy experiments, SOD1‐G93A animals were selected based on their CS [18]. Specifically, for the SOD1‐G93A mild group, only animals with a score of 1–2 were chosen, while for the SOD1‐G93A severe group, animals with a score of 3–4 were selected. WT groups were chosen at 2 months of age and 4 months of age. Only male mice were used. Triceps muscles were dissected and frozen in liquid nitrogen‐cooled isopentane; 8 μm muscle cryosections were used for histology analyses. Cryostat sections were stained with Hematoxylin/Eosin according to classical methods [21, 22]. Briefly, for H&E analysis, cross sections were fixed in 4% formaldehyde at room temperature for 15 min and stained with H&E. Approximately 450 myofibers were measured per WT muscle and 600 myofibers per SOD1‐G93A in the mild and severe stage. Succinic dehydrogenase (SDH) staining was performed according to the Bioptica reagents protocol. Briefly, cryosections were sequentially incubated with a solution containing TRIS aminomethane, hydrochloric acid, cobalt chloride, and thiazolyl blue tetrazolium bromide (MTT) for 45 min at 37°C. Then, sections were dehydrated by a series of increasing alcohol concentrations sequentially: 50% ethanol and picric acid, 70%, 96%, 100% ethanol, and lastly xylene. For NADH diaphorase staining, we followed the Bioptica reagents protocol. Briefly, cryosections were sequentially incubated with a solution containing nitro tetrazolium blue solution, TRIS buffer 0.2 M, pH 7.4, and NADH for 30 min at 37°C. Then, sections were dehydrated by a series of increasing alcohol concentrations sequentially: 50% ethanol and picric acid, 70%, 96%, 100% ethanol, and lastly xylene. The images were acquired by Zeiss microscope and quantification using ImageJ software [23]. Immunostaining and confocal immunofluorescence procedures were performed as previously described [24]. The sections were incubated overnight at +4°C with the following primary antibodies: anti‐TSPO (Abcam Cat# ab109497, RRID:AB_10862345‐Santa Cruz Biotechnology; sc‐518 127), anti‐CD68 (Santa Cruz Biotechnology Cat# sc‐20 060, RRID:AB_627158), anti‐CD86 (rabbit polyclonal antibody, Sigma‐Aldrich; SAB5700710), anti CD206 (BD Biosciences Cat# 555953, RRID:AB_396249) and anti DRP1 (Abcam Cat AMab56788). The sections were then incubated with the corresponding fluorescent‐labeled secondary antibodies, Alexa 488/Alexa 594 conjugated antimouse/antirabbit IgGs (Jackson ImmunoResearch Labs Cat# 711‐545‐152, RRID:AB_2313584, Cat# 705‐585‐147, RRID:AB_2340433). Nuclei were counterstained with Hoechst (Cell Signaling Technology Cat# 4082, RRID:AB_10626776). Images were observed using a Zeiss LSM700 META/laser scanning confocal microscope (Zeiss, Oberkochen, Germany). Single images were taken with a resolution of 1024 × 1024 [25, 26].

### 2.5 The Fluorescence Intensity and Fiber Counting Analysis

Quantification of TSPO, CD68, CD89, DRP1 and CD206 fluorescence intensity on tissue sections at the level of the triceps brachii in terms of pixel intensity value was performed using the image J software (ImageJ, RRID:SCR_003070), as previously described [27]. Briefly, digital images were taken with ×40 objective, and identical laser power settings and exposure times were applied to all the photographs from each experimental set. Images were first thresholder to identify the positive signal; subsequently, the pixels expressing TSPO, CD68, CD86, DRP1 and CD206 were identified [28]. Finally, the number of pixels positive for was measured per microscope field. Images from the same areas of each muscle region were compared. Quantification of TSPO and CD68, CD86, DRP1 and CD206 colocalization was performed by using image J software and the volume colocalization (ImageJ, RRID:SCR_003070), and colocalization thresholders were used. The results were expressed in arbitrary units. *n* = 3/4 mice per treatment group and 3 sections for each genotype. For fiber area quantification, the ImageJ software was used. The fibers were then categorized based on area range (0–100, 100–200, 200–600, 600–1000, 1000–1500, 1500–2000, 2000–3000 μm^2^), and the percentage per image was calculated [29]. A total of 3–4 animals per group were analyzed. To analyze the colocalization index of TSPO with CD86, CD206, and CD68, we used Manders' analysis within ImageJ's JACoP (ImageJ, RRID:SCR_003070). Manders' colocalization coefficients (M1 and M2) were employed to assess the relative contribution of each antigen to colocalized areas. Specifically, M1 represents the sum of red pixel intensities that overlap with a green component, normalized by the total red intensity, while M2 denotes the sum of green pixel intensities overlapping with a red component, divided by the total green intensity. The final colocalization value was obtained by averaging M1 and M2. To enhance accuracy, background correction was applied automatically using Costas' threshold, a built‐in function of the JACoP tool (ImageJ, RRID:SCR_003070).

### 2.6 Statistical Analysis

The nonparametric Mann–Whitney test was used to compare 18F‐DPA‐714 SUVR data among groups. The level of significance was set at *p* ≤ 0.05. For immunohistochemical experiments, statistical significance was assessed by one‐way analysis of variance (ANOVA) followed by Tukey's method of multiple comparison tests (*n* = 3/4 for each group). The level of significance was set at *p* ≤ 0.05. The sample size was calculated using GPower analysis software (G*Power RRID:SCR_013726) to achieve 80% power, with an expected large effect size (0.60) and α = 0.05. For statistical analysis, we used GraphPad Prism 7 program (GraphPad Prism, RRID:SCR_002798).

## 3 Results

### 3.1 PET/CT Images Showed Higher [18F]DPA‐714 SUVR in the Triceps Brachii of SOD1‐G93A Mice in Mild and Moderate–Severe Clinical Stages

**FIGURE 1:**
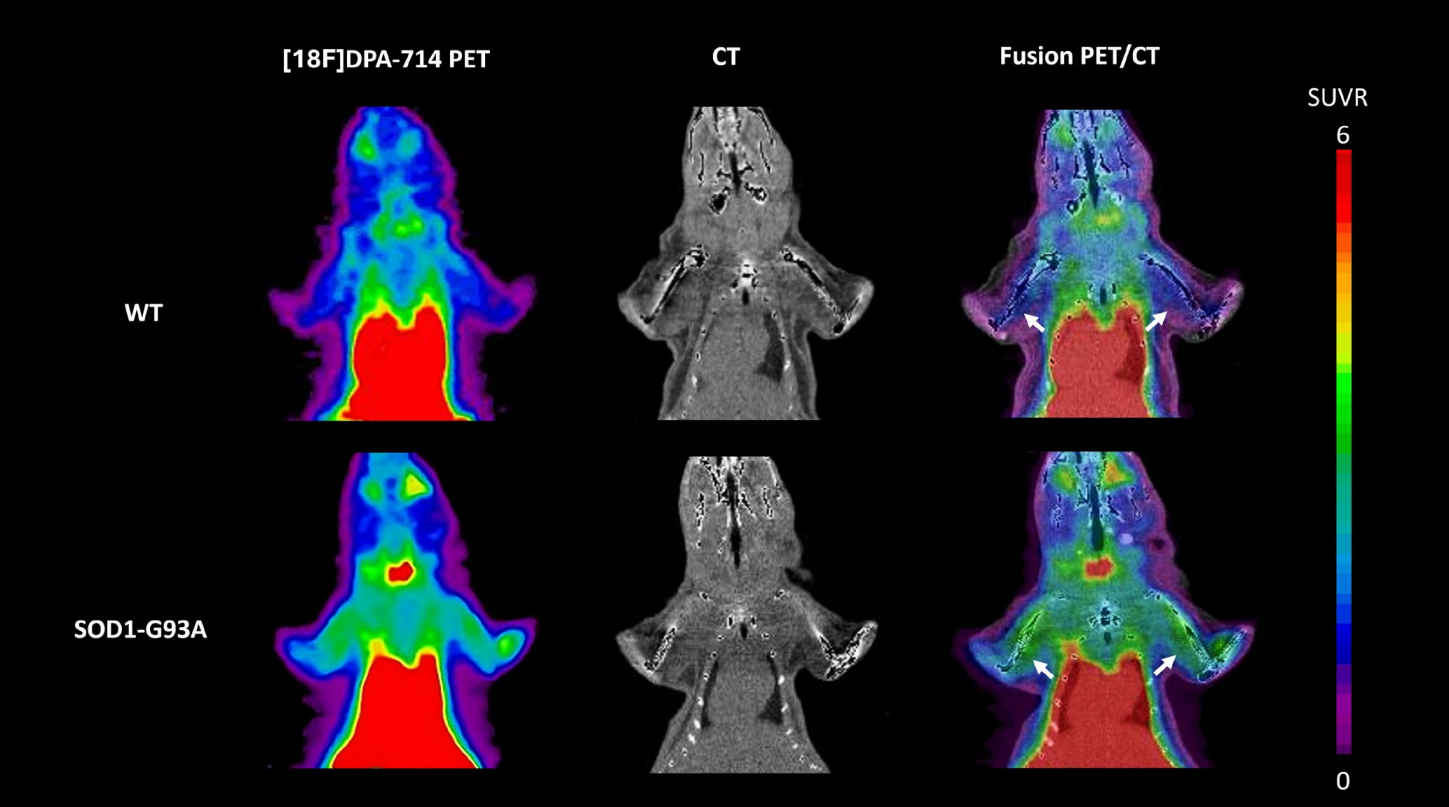
[18F]DPA‐714 PET/CT in ALS mice. [18F]DPA‐714 PET, CT and fusion PET/CT images (coronal plane) of a representative WT mouse and a SOD1‐G93A mouse studied at moderate symptomatic stage (CS 2). PET images represent summed scans obtained between 20 and 50 min postinjection and normalized to SUV values of the associative frontal cortex (SUVR). Increase radiotracer uptake can be observed in the triceps brachii muscles of the SOD1‐G93A as compared to WT. The white arrows indicated the triceps muscles. The SUVR images were scaled to a maximum of 6 and the color scale represents the lower (blue) and the higher (red) SUVR values.

**FIGURE 2:**
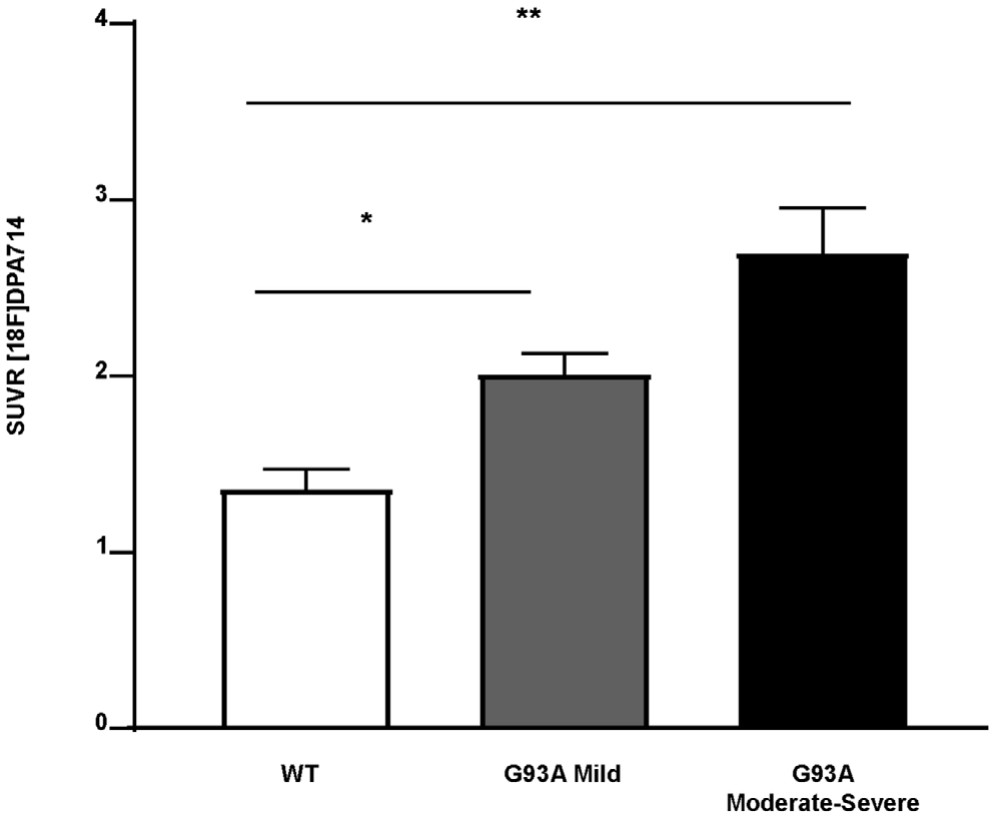
[18F]DPA‐714 SUV ratios. [18F]DPA‐714 SUV ratios (mean ± SD) in the triceps brachii of WT and SOD1‐G93A mice at mild and moderate–severe clinical stages. In both mild and moderate–severe groups averaged [18F]DPA‐714 SUV ratios were significantly increased with respect to WT controls. *p < 0.05; **p < 0.02.

Visual evaluation of PET images showed higher [18F]DPA‐714 SUVR in both triceps brachii in symptomatic G93A mice compared to WT mice (Figure 1). Semiquantitative analysis revealed similar triceps brachii SUVR values in control WT mice at different ages (1.44, 1.684, 1.139, 1.221 at 89, 109, 134, 139 days, respectively; SUVR mean ± SD 1.371 ± 0.244). The triceps brachii SUVR values in symptomatic G93A mice ranged from 1.863 to 3.385 (mean ± SD 2.406 ± 0.571) and were significantly higher than WT (*p* = 0.0028). Averaged [18F]DPA‐714 SUVR values in the triceps brachii were significantly increased in both mild and moderate–severe symptomatic groups as compared with control WT mice (2.025 ± 0.25 and 2.7 ± 0.58 vs. 1.37 ± 0.24 in mild, moderate–severe, and WT groups, respectively, *p* = 0.0286 and *p* = 0.0159, respectively) (Figure 2). There was no significant difference between mean SUVR values in the triceps brachii of mild as compared to moderate–severe symptomatic G93A mice (*p* = 0.0635).

### 3.2 The Triceps of SOD1‐G93A Mice Displayed Skeletal Muscle Fibers Disorganization and High‐Mitochondria‐Content Fibers in Mild and Moderate–Severe Stages of the Disease

**FIGURE 3:**
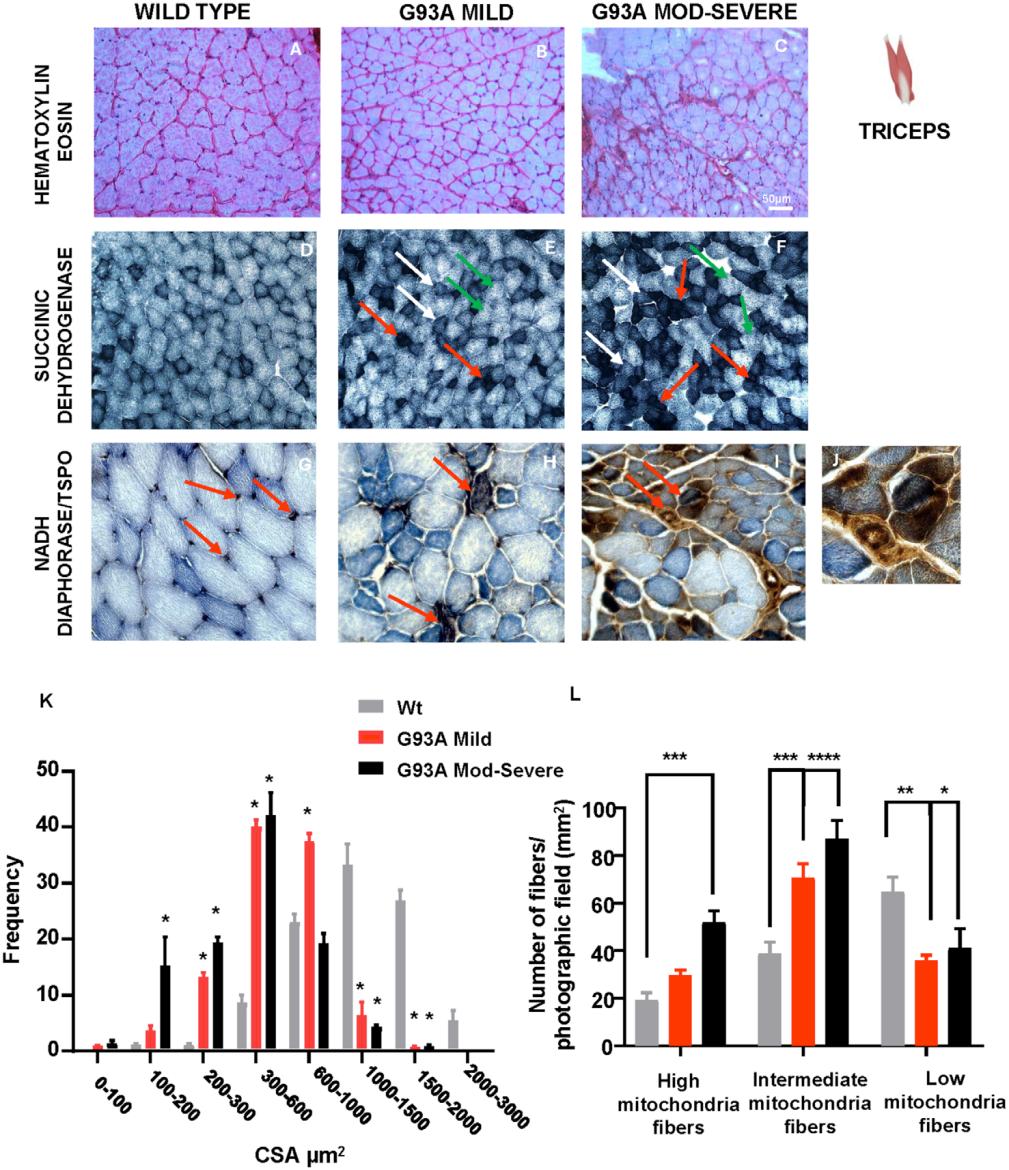
Quantification of muscle damage in the triceps of SOD1‐G93A mice. (A–C) Hematoxylin eosin stained in triceps of WT, SOD1‐G93A mild and SOD1‐G93A moderate–severe mice. (D–F) Succinato Dehydrogenase staining in triceps of WT, SOD1‐G93A mild and SOD1‐G93A moderate–severe mice, green arrows indicate type IIb fibers, white arrows indicate IIa fibers and red arrows indicate type I fibers. (G–J) NADH Diaphorase and TSPO staining, red arrows indicate the TSPO signal. (K) Frequency of Cross section area (um) in triceps of WT, mild SOD1‐G93A and moderate–severe SOD1‐G93A mice. (L) Number of fibers per photographic field (mm2) in triceps of WT, SOD1‐G93A mild and SOD1‐G93A moderate–severe mice. Statistically significant differences among means were determined by one‐way ANOVA followed by Tukey's correction for multiple comparisons test: *p < 0.05, **p < 0.01, ***p < 0.001, ****p < 0.0001. *n* = 3/4 mice per group and 3/4 sections for each genotype.

To assess muscle morphology, hematoxylin–eosin staining was performed on triceps brachii sections from SOD1‐G93A mice in mild and moderate–severe clinical stages and from WT mice. As shown in Figure 3, in mild SOD1‐G93A mice, the triceps muscle displayed evident aspects of degeneration, including slight variability in fiber size, and the appearance of smaller fibers 200–300 μm^2^ in comparison to WT (Figure 3A). Early interstitial infiltration was also observed, likely due to inflammatory cells and connective tissue accumulation, suggesting the onset of a degenerative process (Figure 3B). In contrast, in the moderate–severe stage, muscle structure disorganization becomes more pronounced with increased size variability, a higher proportion of small fibers (100–200 μm^2^), and clear signs of atrophy. Marked increase of interstitial space and a greater infiltration of connective tissue and inflammatory cells were also evident, indicating advanced muscle degeneration. Additionally, some fibers appeared vacuolated or fragmented, a sign of necrosis and severe muscle degeneration (Figure 3C). Quantitative analysis confirmed these changes (Figure 3K). The proportion of small fibers 100–200 μm^2^ significantly increased from ∼3% in WT to ∼5% in mild and ∼15% in severe G93A mice. The most striking difference was observed for fibers in the 300–600 μm^2^ range, highly represented in both mild and moderate–severe SOD1‐G93A mice (∼40% compared to only ∼8% in WT). Larger fibers (1000–1500 μm^2^ and 1500–2000 μm^2^) were markedly and significantly decreased in both mild and moderate–severe groups (∼1%) compared to ∼30% in the WT group, respectively (Figure 3K). To investigate mitochondrial degeneration, SDH staining was performed in the same experimental groups (Figure 3D–F). Both mild and moderate–severe SOD1‐G93A mice showed an increased proportion of high‐mitochondria‐content fibers (type I, see red arrow), intermediate‐mitochondria‐content fibers (IIa, see white arrows), and a decrease in low‐mitochondria‐content fibers (type IIb, see green arrows), indicating a shift in fiber type composition (Figure 3E,F,L). This pattern was even more pronounced in moderate–severe symptomatic SOD1‐G93A mice (Figure 3F,L). Finally, TSPO localization was assessed using a double staining with NADH diaphorase, which distinguishes type 1 fibers (dark blue), type 2 fibers (light blue), and TSPO expression (brown). In WT mice, TSPO was mainly localized in the perimysium (see red arrows, Figure 3G). In mild G93A mice, TSPO expression increased within type I fibers (Figure 3H), and in moderate–severe mice, expression was further increased both within type I fibers and in the perimysium region (Figure 3I,J).

### 3.3 The Triceps Muscle of SOD1‐G93A Mice Showed Alteration in Mitochondrial Fission Either in the Mild and in the Moderate–Severe Stages Paralleled With TSPO Overexpression

**FIGURE 4:**
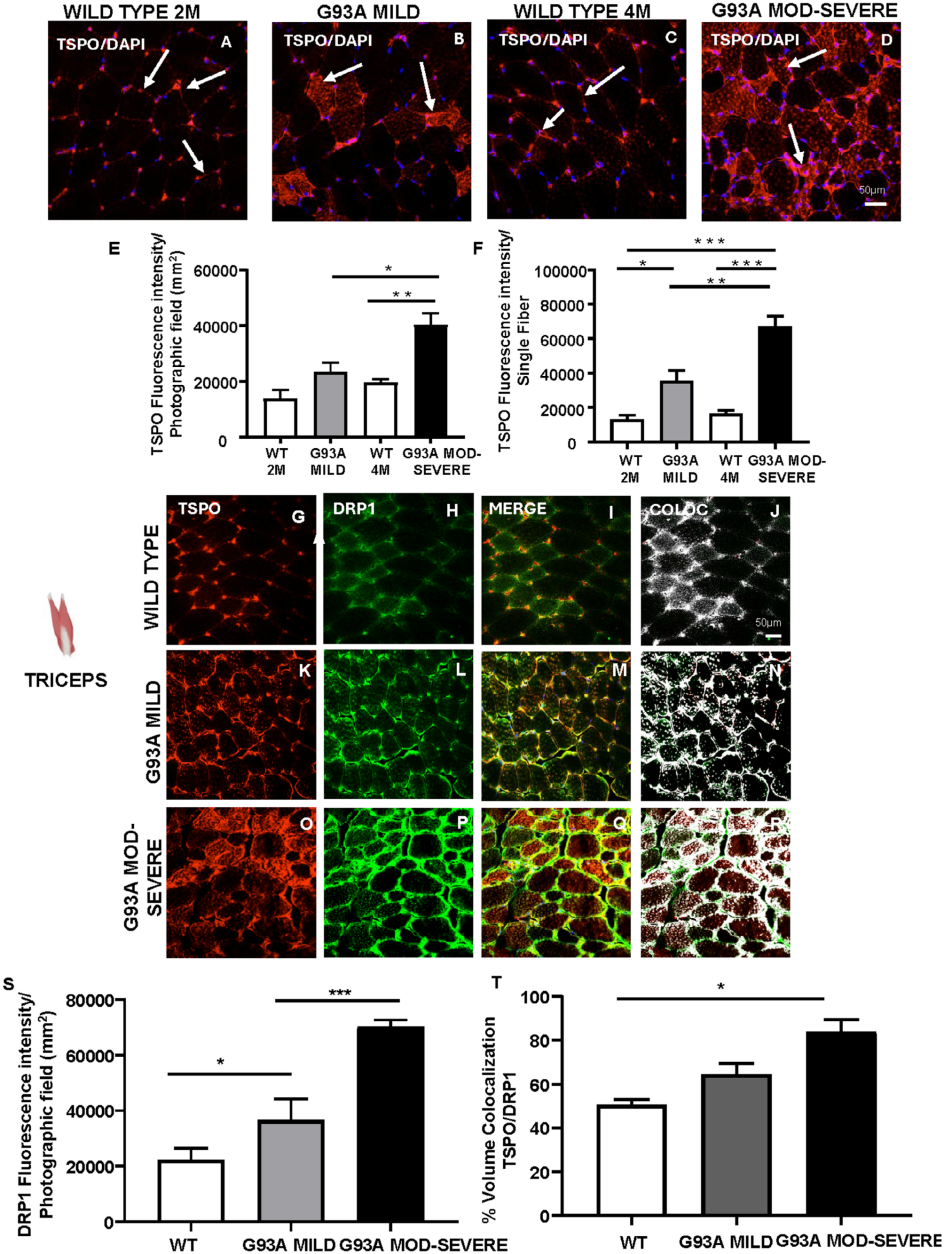
TSPO expression and quantification in the triceps of SOD1‐G93A mice. (A–D) Double labelling of TSPO (red) and Hoechst (blue) in triceps of WT at 2 months of age (A), SOD1‐G93A mild (B), WT at 4 months of age (C) and SOD1‐G93A moderate–severe stage (D). (E) TSPO fluorescence intensity per photographic field (mm^2^) in triceps of WT, SOD1‐G93A mild and SOD1‐G93A moderate–severe mice. (F) TSPO fluorescence intensity per photographic field (mm^2^) in single fiber of WT, SOD1‐G93A mild, and moderate–severe mice. Double labelling of TSPO (red), DRP1 (green), merge (yellow) and colocalization of red and green (white) in triceps of WT (G–J), SOD1‐G93A mild (K–N), and SOD1‐G93A moderate–severe stage (O–R). (S) DRP1 fluorescence intensity per photographic field (mm^2^). (T) Colocalization volume (%) TSPO/DRP1. Statistically significant differences among means were determined by one‐way ANOVA followed by Tukey's correction for multiple comparisons test: *p < 0.05, **p < 0.01, ***p < 0.001. *n* = 3/4 mice per group and 3/4 sections for each genotype.

Immunofluorescence experiments were carried out to assess TSPO localization in the triceps brachii of SOD1‐G93A and WT mice. In WT mice at 2 and 4 months of age, TSPO was expressed in the perimysium and at low levels within muscle (see white arrows, Figure 4A,C,E). In mild symptomatic SOD1‐G93A mice, TSPO expression significantly increased within muscle fiber (see white arrows, Figure 4B,F) but remained comparable to WT levels in the perimysium (see white arrows, Figure 4A,E). In the moderate–severe stage, TSPO expression markedly and significantly increased both in the perimysium and within the muscle fibers compared to WT and the mild stage (see white arrows, Figure 4D–F). To evaluate mitochondrial expression of TSPO, double immunofluorescence staining was performed with dynamin‐related protein 1 (DRP1), a marker of mitochondrial fission and morphology. DRP1 fluorescence intensity significantly increased with disease progression, from mild to moderate–severe stage (Figure 4K–S). TSPO‐DRP1 colocalization also significantly increased with disease severity, significantly in the moderate–severe stage compared to WT, reaching approximately 60% in the mild stage and rising to 80% in the moderate–severe stages compared to WT (Figure 4J,N,R,T).

### 3.4 In the Triceps Skeletal Muscle, TSPO Was Colocalized With CD68, a Marker of Circulating and Tissue‐Resident Macrophages

**FIGURE 5:**
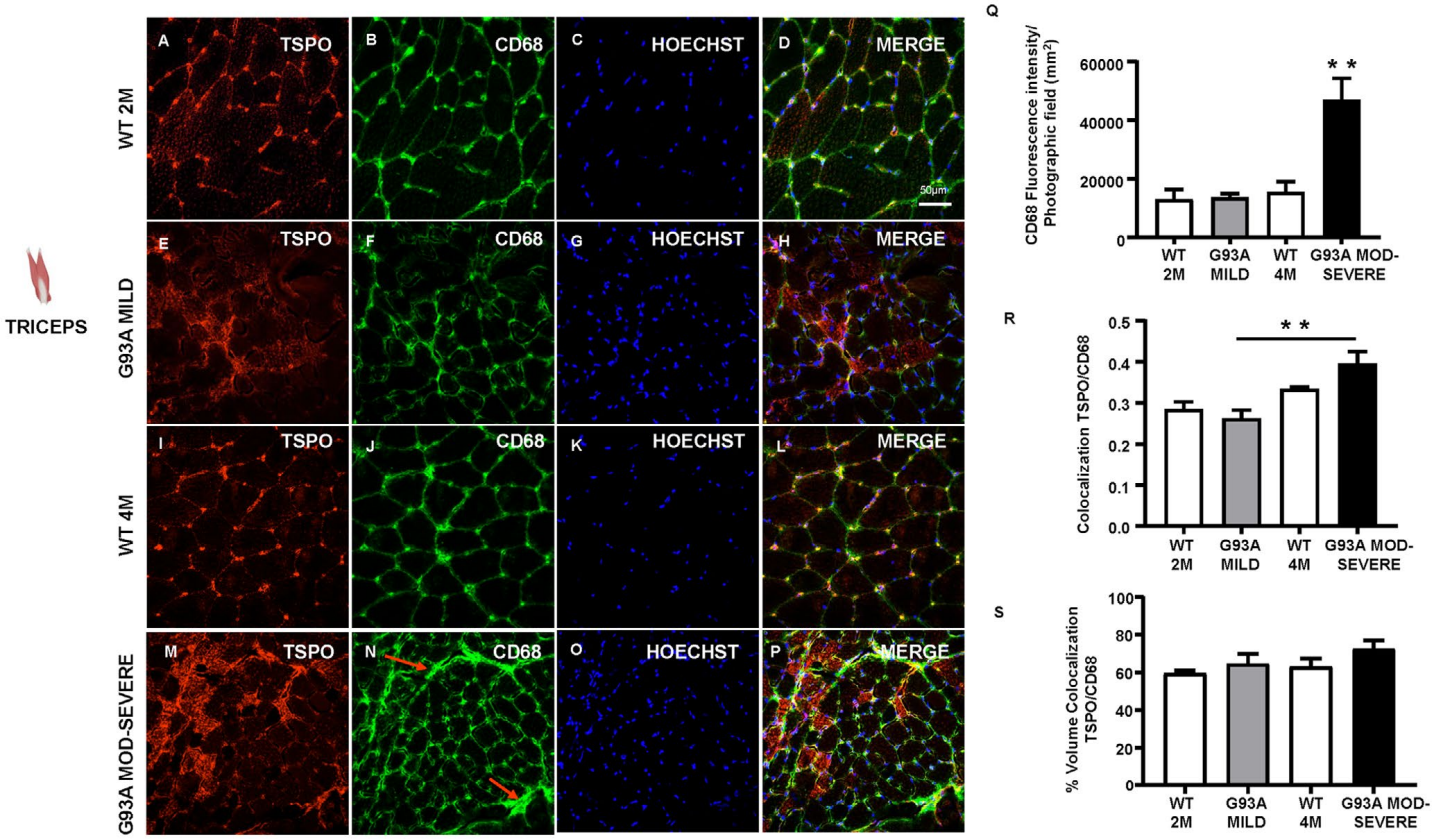
TSPO expression in immune cells of the triceps in SOD1‐G93A mice. Double labelling of TSPO (red), CD68 (green) Hoechst (blue) and merge (yellow) of WT at 2 months of age (A–D), SOD1‐G93A mild (E–H), WT at 4 months of age (I–L) and SOD1‐G93A moderate–severe stage (M–P) in triceps muscle tissue. (Q) CD68 fluorescence intensity per photographic field (mm^2^). (R) Colocalization of TSPO and CD68 (Mander's analysis). (S) Colocalization volume (%) TSPO/CD68. Statistically significant differences among means were determined by one‐way ANOVA followed by Tukey's correction for multiple comparisons test: **p < 0.01. *n* = 3/4 mice per group and 3/4 sections for each genotype.

To better characterize TSPO signaling at the perimysium level, confocal microscopy experiments were performed on the triceps muscles of SOD1‐G93A mice. CD68 fluorescence intensity, a specific marker of M1/M2 macrophages, was significantly elevated in the moderate–severe stage of the disease compared to WT controls (Figure 5N,Q). Furthermore, CD68 localization was restricted to the perimysium region of the fibers in both WT and mild symptomatic mice (Figure 5B,F). In moderate–severe symptomatic G93A mice, CD68 signal increased around the fibers (see red arrows, Figure 5N), reflecting a higher density of macrophages surrounding muscle tissue. Furthermore, TSPO colocalization with CD68 was significantly increased during the moderate–severe stage of the disease (Figure 5R). However, the colocalization volume remained relatively unchanged across the different groups, maintaining a stable level of approximately 60% (Figure 5S).

### 3.5 TSPO Colocalized, in the Triceps Skeletal Muscle, Prevalently With the Protective CD206 Macrophage Marker

**FIGURE 6:**
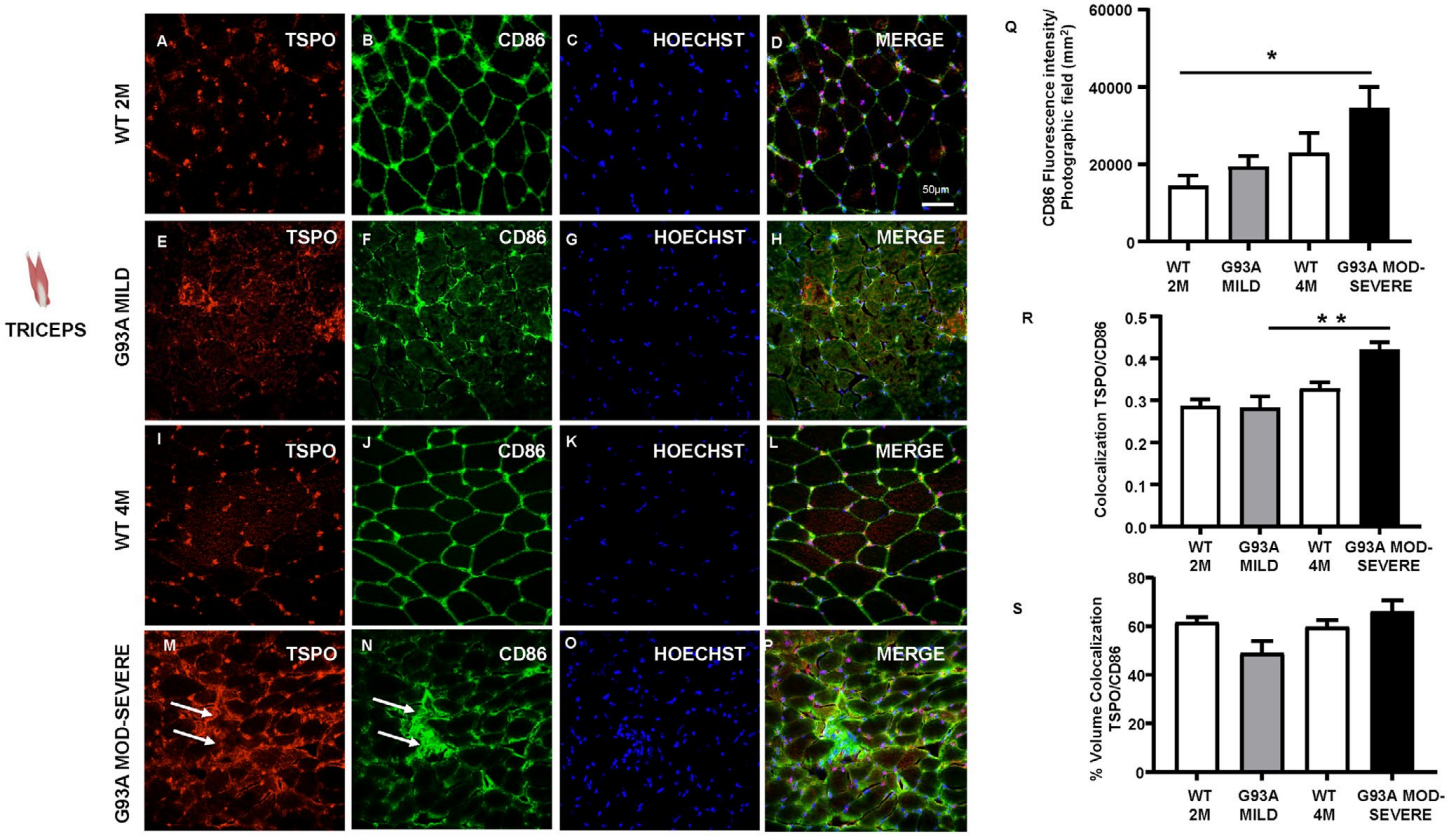
TSPO expression in pro‐inflammatory immune cells of the triceps in SOD1‐G93A mice. Double labelling of TSPO (red), CD86 (green) Hoechst (blue) and merge (yellow) of WT at 2 months of age (A–D), SOD1‐G93A mild (E–H), WT at 4 months of age (I–L) and SOD1‐G93A moderate–severe stage (M–P) in triceps muscle tissue. Q: CD86 fluorescence intensity per photographic field (mm^2^). (R) Colocalization of TSPO and CD86 (Mander's analysis). (S) Colocalization volume (%) TSPO/CD86. White arrows in figure N indicate the accumulation of CD68 staining, and the arrows in figure M indicate the TSPO staining. Statistically significant differences among means were determined by one‐way ANOVA followed by Tukey's correction for multiple comparisons test: *p < 0.05, **p < 0.01. *n* = 3/4 mice per group and 3/4 sections for each genotype.

**FIGURE 7:**
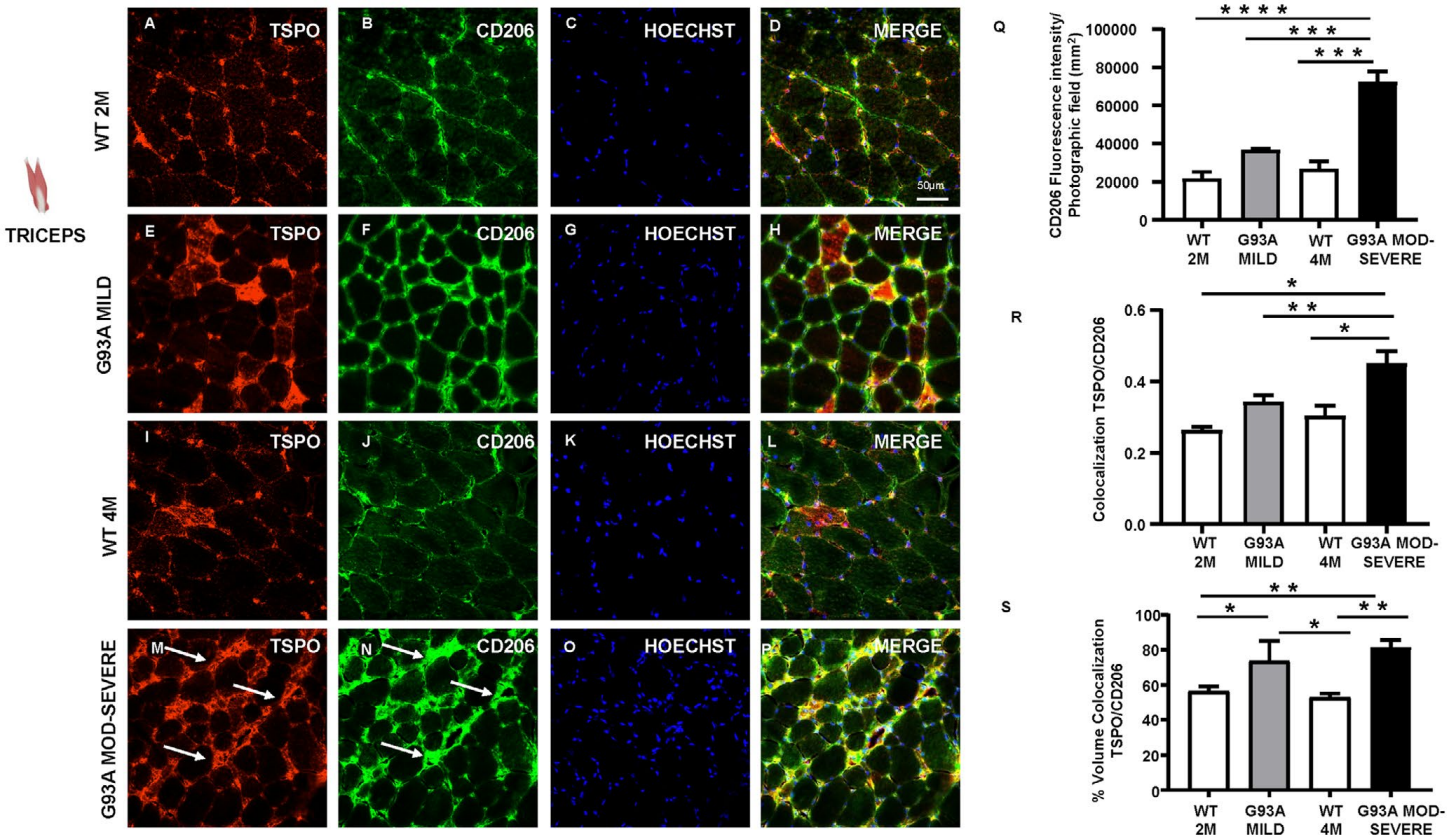
TSPO expression in anti‐inflammatory immune cells of the triceps in SOD1‐G93A mice. Double labelling of TSPO (red), CD206 (green) Hoechst (blue) and merge (yellow) of WT at 2 months of age (A–D), SOD1‐G93A mild (E–H), WT at 4 months of age (I–L) and SOD1‐G93A moderate–severe stage (M–P) in triceps muscle tissue. (Q) CD206 fluorescence intensity per photographic field (mm^2^). (R) Colocalization of TSPO and CD206 (Mander's analysis). (S) Colocalization volume (%) TSPO/CD206. White arrows in figure N indicate the accumulation of CD206 staining, and the arrows in figure M indicate the TSPO staining. Statistically significant differences among means were determined by one‐way ANOVA followed by Tukey's correction for multiple comparisons test: *p < 0.05, **p < 0.01, ***p < 0.001, ****p < 0.0001. *n* = 3/4 mice per group and 3/4 sections for each genotype.

To determine whether TSPO was more prevalent in inflammatory or protective macrophages, confocal microscopy experiments were performed using CD86 and CD206. As shown in Figure 6, CD86 fluorescence intensity significantly increased only in the moderate–severe stage of the disease compared to WT (Figure 6Q). In contrast, CD86 expression remained perivascular across all analyzed groups; in the moderate–severe symptomatic mice, it formed aggregates that infiltrated the muscle fibers (see red arrows, Figure 6N). TSPO colocalization with CD86 showed a significant increase in the moderate–severe stage; however, the colocalization volume remained stable at approximately 60% across all groups (Figure 6R). Notably, TSPO expressions within the CD86‐positive aggregates appeared low or nearly absent (see white arrows, Figure 6M,N). Regarding the CD206 marker, fluorescence intensity increased significantly only in the moderate–severe stage compared to WT and mild groups (Figure 7Q). CD206 distribution remained confined at the perimysium level across all experimental groups (Figure 7A–P); nevertheless, in the SOD1‐G93A moderate–severe group, CD206 signal formed aggregates adjacent to the muscle fibers (see white arrows, Figure 7M,N). TSPO colocalization with CD206 was already elevated in the mild stage and became significantly higher in the SOD1‐G93A moderate–severe stage (Figure 7R). This trend was evident in the colocalization volume graph, where colocalization significantly increased in the SOD1‐G93A moderate–severe group, reaching a level of up to 80% (Figure 7S). Moreover, within CD206‐positive aggregates, TSPO colocalized almost completely (see red arrows, Figure 7M,N).

## 4 Discussion

The results of this study suggested that TSPO expression significantly increases in the triceps brachii skeletal muscle of symptomatic SOD1‐G93A mice, as measured in vivo with [18F]DPA‐714 PET/CT and by immunohistochemistry and immunofluorescence, since the mild clinical stages. Moreover, immunofluorescence analysis revealed that both infiltrated macrophages and muscle fibers contribute to the increased expression of TSPO in the skeletal muscle of this ALS model. Specifically, TSPO colocalized with DRP1, a marker of mitochondrial fission, predominantly in type I muscle fibers starting from the mild stage of ALS. In moderate–severe stage, TSPO expression extended into the perimysium, where it colocalized predominantly with CD206, a marker of M2 macrophages. TSPO expression has been extensively investigated in vivo using PET imaging in the central nervous system (CNS) of ALS patients, and to a lesser extent, in preclinical ALS models. Increased binding of both first‐ and second‐generation TSPO radioligands has been reported in the motor cortex, thalamus, and pons of ALS patients [30] as well as in the brainstem of symptomatic transgenic SOD1‐G93A mice [18] and rats [31]. The increased TSPO expression in the CNS of ALS patients and in animal models is mainly associated with microglia activation and/or increased microglia density [32]. Interestingly, activation of the peripheral immune system is also implicated in the pathophysiology of ALS, and infiltration of macrophages and other immune cells has been reported in the peripheral nerves and skeletal muscles of ALS transgenic mice [7, 10, 32, 33, 34], and in ALS patients [35]. Moreover, TSPO is also expressed by peripheral immune cell types [36] including macrophages [37]. Importantly, TSPO is localized on the outer mitochondrial membrane and plays a key role in mitochondrial function, in particular in the regulation of cholesterol transport [38] that is altered in ALS patients [12, 39]. Epidemiological studies investigating the role of cholesterol in ALS have yielded conflicting results, making it unclear whether cholesterol acts as a protective or detrimental factor in disease progression [1, 2, 3, 4, 40, 41, 42, 43]. Importantly, sex‐dependent differences in cholesterol metabolism, influenced by hormonal and genetic factors, have been reported. For instance, in ALS patients, elevated HDL (high‐density lipoprotein) levels have been associated with a poorer prognosis across both sexes, whereas low LDL (low‐density lipoprotein) levels appear to be a negative prognostic factor only in females. These findings underscore the need to consider sex as a critical variable when evaluating the impact of cholesterol on ALS pathogenesis [44]. Therefore, TSPO may represent an attractive target for the study of skeletal muscle involvement in ALS pathophysiology at two levels: (a) as a marker of muscle inflammation and (b) as an indicator of mitochondrial alterations. Our results demonstrated that increased TSPO expression in the triceps muscles could be assessed with [18F]DPA714 PET in mild symptomatic SOD1‐G93A mice (+47.8%), when their CS ranged between 1 and 1.5. The [18F]DPA714 SUVR values further increased in the moderate–severe stages (+97%). These in vivo findings are supported by the results of our immunohistochemical analyses. In fact, confocal microscopy experiments showed that increased TSPO expression predominantly colocalized, since the mild stage, with the so‐called “protective” CD206 macrophage marker rather than CD86, the protein marker primarily expressed on antigen‐presenting cells (APCs) like B cells, dendritic cells, and macrophages. Quantitative analysis confirmed these findings, showing 80% colocalization of TSPO with CD206, compared to 60% with CD86 at moderate–severe disease stages. The increased expression of inflammatory macrophage markers observed in our study is in part in line with previous data in SOD1‐G93A murine models showing a significant increase staining of CD68 and CD11b in the tibialis anterior muscle end‐stage of SOD1‐G93A rats [10]. However, a more abundant CD206+ cells in SOD1‐G93A quadriceps muscles at the advanced stage was observed [7]. Furthermore, in a recent study, Marini et al. reported that TSPO expression in the skeletal muscle of SOD1‐G93A mice was primarily localized in myocytes, due to the absence of detectable inflammatory marker CD68 signals in the quadriceps muscle [45]. In contrast, our data revealed a clear up‐regulation of CD68, CD86, and CD206 in the triceps muscle during the moderate–severe stage of the disease. These discrepancies may be explained by the fact that Marini et al. used conventional immunohistochemistry, whereas our findings were obtained using confocal microscopy, a technique with significantly higher sensitivity and resolution. Accordingly, our results support the hypothesis that increased TSPO expression in the skeletal muscle of SOD1‐G93A is related to increased inflammation mainly mediated by so‐called “protective phenotype,” since the mild stages, and suggest that TSPO may play a role in regulating an anti‐inflammatory and potentially protective macrophage phenotype in the context of ALS along the different disease stages. Notably, we found that, already during the mild stages of the disease, the triceps muscles of SOD1‐G93A mice exhibited clear signs of muscle fiber degeneration. At this stage, muscle fiber degeneration is characterized not only by a reduction in cross‐sectional area but also by distinct structural abnormalities, including sarcolemma fragmentation [46], basal lamina disruption [47], and central nucleation features indicative of ongoing regenerative processes in response to prior damage [48]. Furthermore, electron microscopy experiments have demonstrated changes in skeletal muscle mitochondrial ultrastructure, with elongated and fragmented mitochondria in SOD1‐G93A mice at the early stage of the disease, around 80 days of age [49]. In parallel with fiber size reduction, we also observed a significant rise in fibers with intermediate mitochondrial content, typically associated with type IIa fibers [50, 51]. This shift may be a compensatory response to early neuromuscular stress or partial denervation, as IIa fibers are metabolically more adaptable than fast‐glycolytic IIb fibers [52]. Their increased oxidative capacity and mitochondrial content could help them better cope with altered energetic demands and oxidative stress [53]. As the disease advances to its late stages, muscle fiber degeneration becomes more pronounced. We observed a clear reduction in low mitochondrial content type IIb fibers and a relative increase in oxidative, high mitochondrial content type I fibers. This shift reflects the selective vulnerability of fast‐twitch, glycolytic fibers, which degenerate earlier due to their lower mitochondrial efficiency and reduced capacity to handle oxidative stress [54, 55]. These fibers often show early signs of mitochondrial swelling and excessive ROS production, hallmarks of dysfunction. In contrast, type I fibers, known for their endurance and resistance, appear to survive longer, likely thanks to their higher mitochondrial density and more robust antioxidant defenses [56]. These findings align with previous work reporting a progressive reprogramming of muscle fiber composition from fast‐ to slow‐twitch profiles during ALS progression [55]. Such changes are closely linked to mitochondrial alterations, which are exacerbated by muscle‐specific overexpression of SOD1‐G93A [57]. The increased TSPO expression in these fibers, coupled with its enhanced colocalization with DRP1 suggests a potential protective or compensatory mechanism aimed at preserving mitochondrial structure and function under chronic stress. These findings are in line with previously reported alterations in mitochondrial ultrastructure and increased fluorodeoxyglucose uptake in skeletal muscle of SOD1‐G93A mice [7]. A particularly interesting finding emerged when analyzing TSPO expression. We observed that in wild‐type animals, around 40% of the TSPO signal colocalizes with DRP1, a key protein involved in mitochondrial fission. This colocalization increases significantly in SOD1‐G93A mice, reaching approximately 60% in the mild stage and up to 80% in the moderate–severe stage. This progressive association points toward the role of TSPO in mitochondrial dynamics under pathological stress, possibly promoting fission as an adaptive response. This observation adds weight to the hypothesis that TSPO might serve not only as a marker but also as a modulator of mitochondrial health in degenerating muscle. The main limitations of this study are the small number of animals used for PET/CT analysis and the fact that only male mice were used. In addition, there is a lack of more comprehensive investigation of other muscles affected by the disease, such as the quadriceps and soleus, which exhibit significant differences in both mitochondrial bioenergetics and fiber composition. These limitations need to be addressed in future studies. Overall, the results of the present study support the potential of TSPO‐PET as a noninvasive diagnostic imaging biomarker for early detection and monitoring of skeletal muscle degeneration and highlight the potential role of TSPO as a druggable target in ALS.

## Author Contributions

Serenella Anzilotti: writing, investigation, methodology, and data curation. Nunzia De Iesu: investigation, methodology. Sara Gargiulo: writing and editing, formal analysis, and methodology. Noemi Di Muraglia: investigation, methodology. Annunziata Gaetana Cicatiello, Monica Dentice, Mariarosaria Panico, and Sandra Albanese: writing and editing. Lucio Annunziato, Marco Salvatore: founding acquisition and writing. Giuseppe Pignataro and Sabina Pappatà: writing, investigation, methodology, and founding acquisition.

## Conflicts of Interest

The authors declare no conflicts of interest.

## Data Availability

The data supporting the findings and conclusions of this study are available upon request to the corresponding author, S.A., S.P. and S.A.
